# Circulating antibodies to α-enolase and phospholipase A_2_ receptor and composition of glomerular deposits in Japanese patients with primary or secondary membranous nephropathy

**DOI:** 10.1007/s10157-016-1235-2

**Published:** 2016-02-01

**Authors:** Yukihiro Kimura, Naoto Miura, Hanna Debiec, Hiroyuki Morita, Harutaka Yamada, Shogo Banno, Pierre Ronco, Hirokazu Imai

**Affiliations:** 1 Division of Nephrology and Rheumatology, Department of Internal Medicine, Aichi Medical University School of Medicine, Nagakute, Aichi 480-1195 Japan; 2 INSERM UMR_S 1155, UPMC Univ-Paris 6, Assistance Publique–Hôpitaux de Paris (AP-HP), Tenon Hospital, Paris, France; 3 Department of Internal Medicine, Kawana Hospital, Nagoya, Japan

**Keywords:** α-Enolase, Phospholipase A_2_ receptor, Membranous nephropathy

## Abstract

**Background:**

Phospholipase A_2_ receptor (PLA_2_R) is recognized as a target antigen in primary membranous nephropathy (MN); Anti-α-enolase antibody in primary and secondary MN has been proposed, however, little is known about the potential contribution of α-enolase to the pathogenesis of MN.

**Methods:**

We evaluated circulating antibodies to α-enolase by a dot blotting system and PLA_2_R by indirect immunofluorescence, and glomerular deposition of these proteins in 25 patients with primary MN, 20 patients with secondary MN, 44 patients with collagen disease or severe infection, 60 patients with nephritis (each ten patients of IgA nephropathy, focal segmental gloemrulosclerosis, minimal change nephrotic syndrome, membranoproliferative glomeurlonephritis, diabetic glomerulosclerosis, and tubulointerstitial nephritis) as disease control, and 20 healthy subjects.

**Results:**

In primary MN, 18 of 25 sera (72 %) showed anti-α-enolase antibody (IgG1 and IgG4, 11 pts; IgG4 alone, six pts; IgG1 alone, one pt). In secondary MN, 15 of 20 sera (75 %) contained anti-α-enolase antibody (IgG1 and IgG3, 13 pts; IgG3 alone, two pts). No circulating anti-α-enolase antibody was found in 44 collagen diseases or septic patients, 60 nephritis without MN, and 20 healthy subjects. Twelve of 25 sera (48 %) from patients with primary MN were positive for anti-PLA_2_R antibody, whereas all patients with secondary MN were negative. Eight of the 12 PLA_2_R-positive patients (67 %) with primary MN also had anti α-enolase antibody. Although PLA_2_R antigen was present in a subepithelial pattern in 10 of 19 (52 %) patients with primary MN, α-enolase was never detected in glomerular deposits in 19 and ten patients with primary and secondary MN, respectively.

**Conclusions:**

Circulating anti-α-enolase antibodies are highly present in both primary and secondary MN (about 70 %, respectively), while anti-PLA_2_R antibodies are specific for primary MN (48 %) with a prevalence apparently lower in the Japanese population than in Chinese and Caucasian populations. The absence of α-enolase from subepithelial immune deposits suggests that anti-α-enolase antibodies do not contribute directly to immune-deposit formation, although they may have other pathogenic effects.

**Electronic supplementary material:**

The online version of this article (doi:10.1007/s10157-016-1235-2) contains supplementary material, which is available to authorized users.

## Introduction

Membranous nephropathy (MN)—a major glomerular disease and common cause of adult nephrotic syndrome—is characterized by glomerular subepithelial IgG deposits [1]. Primary MN is predominantly associated with glomerular deposition of the IgG4 subtype although variable amounts of IgG1 were also detected in immune deposits [2–6], whereas secondary MN is characterized by prevailing deposits of IgG1, IgG2, and IgG3 [6–8]. During the past decade, several breakthroughs have occurred with the identification of several candidate human antigens in MN. In infants with MN, Debiec et al. detected anti-neutral endopeptidase antibodies that were produced by mothers who lacked this enzyme [9, 10]. Beck et al. demonstrated that IgG4 antibodies specific for M-type phospholipase A_2_ receptor (PLA_2_R) were present in glomerular eluates and serum from adult patients with primary MN [11]. Other candidate autoantigens in patients with primary MN include superoxide dismutase 2 (SOD2), aldose reductase [12] and α-enolase [13–15]. Debiec et al. reported that some patients who develop MN in early childhood had circulating anti-bovine serum albumin (BSA) antibodies and cationic BSA as a component of glomerular immune deposits [16]. Recently, Anti-thrombospondine type-1 domain-containing 7A antibody was reported in 10 % of primary MN patients [17].

Interestingly, unlike other antibodies identified in patients with autoimmune MN, anti-α-enolase antibodies have been found in both primary and secondary MN [13, 14, 18]. In the present study, we examined the potential relationship between circulating anti-α-enolase antibodies and anti-PLA_2_R antibodies, and the glomerular deposition of α-enolase and PLA_2_R proteins in Japanese patients with primary and secondary MN.

## Materials and methods

This study was approved by the Ethics Committee at Aichi Medical University (10–127).

### Patients’ characteristics

We included Japanese patients with MN who were admitted to Aichi Medical University Hospital and affiliated hospitals between 2003 and 2011. Diagnosis of MN was based on light microscopy, immunofluorescence, and electron microscopy analyses of kidney biopsy specimens [19]. Primary MN was defined according to morphologic criteria after excluding known underlying diseases and drug exposure. Sera from 25 patients with primary MN, 20 patients with secondary MN, 44 patients with collagen diseases or severe infection, 60 patients with nephritis without MN (each ten patients of IgA nephropathy, focal segmental gloemrulosclerosis, minimal change nephrotic syndrome, membranoproliferative glomeurlonephritis, diabetic glomerulosclerosis, and tubulointerstitial nephritis) as disease control, and 20 healthy subjects were stored at −80 °C until use. Sera from all patients with MN were obtained before steroid and immunosuppressive treatment with the approval of the institutional ethics committee. Patients’ characteristics are summarized in Tables 1 and 2. Complete remission was defined as urinary protein excretion of less than 300 mg/day as a result of therapeutic intervention such as prednisolone with or without immunosuppressive drugs; spontaneous remission was defined as complete remission in a patient receiving anti-platelets or anti-hypertensive drugs only. Partial remission was defined as proteinuria <3.5 g/day with a decrease of proteinuria <50 % from baseline and stable renal function.

**Table 1:** Characteristics of patients with primary membranous nephropathy

	Age	Sex	Stage of MN	Amount of urinary protein (g/day)	Serum albumin (g/dl)	Serum creatinine (mg/dl)	Anti-α- enolase antibody	IgG subclass	PLA2R-AB	IgG subclass	PLA2R in biopsy	Treatment
MN-1	52	F	I	2.60	3.0	0.52	Pos	1 + 4	Pos	4	Pos	PSL + MZR
MN-2	74	M	I to II	4.05	1.6	0.75	Pos	4	Pos	1 + 4	Pos	PSL + MZR
MN-3	18	M	I	1.41	3.5	0.38	Pos	1 + 4	Neg	Neg	Na	Dipyridamole
MN-4	77	M	I	3.04	1.4	1.00	Neg	Neg	Pos	4	Na	Unknown
MN-5	43	M	I to II	6.86	2.8	0.69	Pos	1 + 4	Neg	Neg	Na	PSL + MZR
MN-6	65	M	III	0.58	4.1	0.85	Pos	1	Neg	Neg	Na	PSL + MZR
MN-7	70	M	II to III	4.46	2.4	0.85	Pos	1 + 4	Pos	4	Na	PSL + MZR
MN-8	75	F	II	3.00	2.4	1.05	Pos	1 + 4	Pos	1 + 4	Na	Unknown
MN-9	62	F	I to II	0.62	2.9	0.48	Pos	1 + 4	Neg	Neg	Pos	ARB
MN-10	65	M	I	4.16	2.4	0.88	Neg	Neg	Pos	4	Pos	PSL + MZR
MN-11	62	M	II	3.40	2.7	0.79	Pos	4	Pos	4	Pos	PSL + MZR
MN-12	52	F	I	5.00	1.9	0.59	Neg	Neg	Neg	Neg	Neg	PSL + MZR
MN-13	77	F	I	3.15	1.3	0.57	Neg	Neg	Neg	Neg	Neg	ARB
MN-14	61	F	I	8.00	1.6	0.45	Neg	Neg	Pos	4	Pos	Unknown
MN-15	69	M	I to II	4.44	2.9	1.54	Pos	1 + 4	Neg	Neg	Neg	PSL + MZR
MN-16	58	F	I to II	1.10	3.8	0.44	Pos	4	Pos	4	Pos	PSL + MZR
MN-17	33	M	I	1.16	3.2	0.72	Neg	Neg	Neg	Neg	Neg	Unknown
MN-18	47	F	I to II	2.62	2.7	0.77	Pos	1 + 4	Neg	Neg	Neg	PSL + MZR
MN-19	69	F	I	8.47	2.1	0.98	Pos	1 + 4	Neg	Neg	Neg	PSL + MZR
MN-20	57	F	I	1.24	1.8	0.97	Neg	Neg	Pos	1 + 4	Pos	PSL + MZR
MN-21	73	M	I to II	6.41	3.0	1.04	Pos	4	Pos	4	Pos	ARB
MN-22	56	M	II to III	0.30	3.4	0.68	Pos	1 + 4	Neg	Neg	Neg	Unknown
MN-23	65	M	I	0.42	3.6	0.77	Pos	1 + 4	Neg	Neg	Neg	None
MN-24	68	M	II	0.67	3.3	0.67	Pos	4	Neg	Neg	Neg	ARB
MN-25	77	M	I	1.82	2.8	0.92	Pos	4	Pos	4	Pos	ARB

*ARB* angiotensin II receptor blocker, *MN* menbranous nephropathy, *MZR* mizoribin, *na* not available, *PSL* prednisolone, *PLA2R AB* anti-PLA2R antibody

**Table 2:** Characteristics of patients with secondary membranous nephropathy

	Age	Sex	Stage of MN	Amount of urinary protein (g/day)	Serum albumin (g/dl)	Serum creatinine (mg/dl)	Anti-α- enolase antibody	IgG subclass	PLA2R-AB	IgG subclass	PLA2R in biopsy	Treatment
LN-1	46	F	lupus V	2.19	2.9	0.57	Pos	1 + 3	Neg	Neg	Na	PSL + MZR
LN-2	54	F	lupus V	4.32	2.5	0.69	Neg	Neg	Neg	Neg	Na	PSL + MZR
LN-3	58	M	lupus V	3.23	2.4	0.74	Neg	Neg	Neg	Neg	Na	PSL
LN-4	57	M	lupus V	3.15	4.0	0.93	Neg	Neg	Neg	Neg	Na	PSL + TAC
LN-5	24	F	lupus V	11.10	1.1	0.44	Pos	1 + 3	Neg	Neg	Neg	PSL
LN-6	40	F	lupus V	5.69	2.5	0.50	Pos	1 + 3	Neg	Neg	Neg	PSL + TAC
LN-7	18	F	lupus V	3.34	1.7	0.85	Neg	Neg	Neg	Neg	Na	PSL
LN-8	35	F	lupus V	0.52	3.7	0.53	Pos	1 + 3	Neg	Neg	Neg	None
LN-9	49	M	lupus V	1.44	2.6	1.00	Pos	1 + 3	Neg	Neg	Neg	PSL + MZR
LN-10	42	F	lupus V	3.07	2.4	0.69	Pos	3	Neg	Neg	Neg	PSL
LN-11	19	F	lupus V	0.13	4.4	0.81	Pos	1 + 3	Neg	Neg	Neg	Unknown
LN-12	42	F	lupus V	1.23	2.6	0.48	Pos	1 + 3	Neg	Neg	Neg	PSL + MZR
LN-13	47	F	lupus V	0.10	4.3	0.52	Pos	1 + 3	Na	Na	Neg	PSL
BUC-1	56	F	I to II	3.08	3.2	0.46	Pos	1 + 3	Neg	Neg	Na	PSL + MZR
BUC-2	52	M	I	2.46	2.4	0.58	Pos	1 + 3	Neg	Neg	Na	PSL
BUC-3	69	M	I to II	8.94	3.2	0.99	Pos	3	Neg	Neg	Na	PSL
BUC-4	70	M	I	2.00	3.1	0.78	Pos	1 + 3	Neg	Neg	Na	PSL + ARB
BUC-5	63	M	I to II	3.68	1.9	0.65	Pos	1 + 3	Neg	Neg	Na	ARB
BUC-6	53	M	I	0.30	3.7	0.50	Pos	1 + 3	Neg	Neg	Neg	PSL
BUC-7	79	M	I	9.50	1.6	0.72	Neg	Neg	Neg	Neg	Neg	Unknown

*ARB* angiotensin II receptor blocker, *BUC* bucillamine, *LN* lupus nephritis, *MN* menbranous nephropathy, *MZR* mizoribin, *na* not available, *PSL* prednisolone, *TAC* tacrolimus

### Preparation of α-enolase deletion mutants

We obtained sequence encoding full-length human α-enolase (433 amino acids; GenBank AK315417, Ensembl: ENSG00000074800). As a result of post-translational modification, human α-enolase is a 47-kDa glycoprotein. Complementary DNA (cDNA) cloning and production of fusion proteins were described elsewhere [20]. Briefly, full-length and truncated cDNA encoding human α-enolase was amplified in polymerase chain reactions, and ligated to sequence encoding glutamine S-transferase (GST) (GE Healthcare Bio-Sciences Corp., Piscataway, NJ). DNA was cloned into pGEX plasmids and transformed into JM109 cells (Promega, USA), and protein expression was induced using isopropyl-β-d-thiogalactopyranoside. After protein extraction from JM109 cells, tagged proteins were affinity purified using glutathione-Sepharose 4B (Amersham Pharmacia Biotech).

### Anti-α-enolase antibody by western and dot blot analysis

Nitrocellulose membranes (GE Healthcare, UK) were washed in PBS (Wako Pure Chemical Industries, Japan) containing 10 μg/ml recombinant α-enolase protein and 5 % (w/v) skim milk (BD Difco, USA) for 60 min at room temperature in non-reducing conditions. The membrane was washed three times with PBS containing 0.05 % Tween (Katayama Chemical Industries, Japan), and incubated with patient serum at a dilution of 1:200. After three washes in PBS containing 0.05 % Tween, the membrane was incubated with horseradish peroxidase–conjugated anti-human IgG antibodies at a dilution of 1:1000 (Sigma-Aldrich, USA), and peroxidase–conjugated mouse monoclonal antibodies to human IgG1, IgG2, IgG3, IgG4 at a dilution of 1:500 (Invitrogen, USA). After three washes, reaction product on the membrane was visualized using an enhanced chemiluminescence system (ChemiLumit Kit, GE Health Care, UK). Photographic images were obtained using an LAS-1000 system (FujiFilm Co., Japan). Image Reader Lite for LAS-1000 plus Ver. 1.3 (FujiFilm Co.) was used to capture the images, which were edited using Adobe Photoshop, when necessary.

### Anti-PLA_2_R antibody by indirect immunofluorescence

Anti-PLA_2_R specific autoantibody titers were measured as previously described based on indirect immunofluorescence in HEK 293 cells that were transiently transfected with full-length cDNA encoding PLA_2_R (Euroimmun) [21]. Antibody positivity was defined as positive staining at serum dilutions of at least 1/10. Negative results for anti- PLA_2_R antibodies were defined as an absence of detectable signals at an antibody dilution of 1/10.

### Glomerular deposition of α-enolase and PLA_2_R proteins

α-Enolase and PLA_2_R were detected in paraffin-embedded native biopsies under a confocal microscope using affinity-purified specific anti-rabbit α-enolase (AbD serotec) and PLA_2_R antibodies (Atlas Antibodies, Stockholm, Sweden) followed by goat Alexa 488 conjugated anti-rabbit IgG Fab fragments (Molecular Probes, Eugene, OR, USA) as previously described [21]. Staining with only secondary antibodies produced negative results for all biopsies.

### Statistical analysis

Data were analyzed using Microsoft Excel software. Normally distributed variables were described as mean standard deviation and compared across primary and secondary MN using analysis of variance. Age, amount of urinary protein, and serum creatinine were compared using Student’s *t* test. Mann–Whitney *U* tests (nonparametric) were used to compare results for anti-α-enolase antibody positivity in patients with MN and control subjects. All *p* values are two tailed, with <0.05 considered statistically significant.

## Results

### Patients’ characteristics

The study comprised 25 patients with primary MN, and 20 patients with secondary MN (seven patients had bucillamine-induced nephropathy and 13 patients had lupus nephritis World Health Organization type V). Patients’ characteristics are summarized in Tables 1 and 2. The mean age of the patients with primary and secondary MN was 61.0 ± 14.4 and 48.7 ± 16.3 years, respectively (*p* < 0.01). The mean serum creatinine level was 0.77 ± 0.25 and 0.67 ± 0.18 mg/dl (*p* = 0.07), and mean proteinuria was 3.16 ± 2.39 g/day and 3.47 ± 3.13 mg/day (*p* = 0.6), at the time of diagnosis in primary and secondary MN, respectively. Treatment modalities were not known in seven patients. One patient received dipyridamole alone, 29 patients were treated with prednisolone with or without other immunosuppressants including Mizoribine (an immunosuppressive agent used in Japan), six patients were treated with angiotensin II receptor blocker (ARB) alone, and two patients received no therapy.

### Detection of anti-α-enolase antibody

**Fig. 1:**
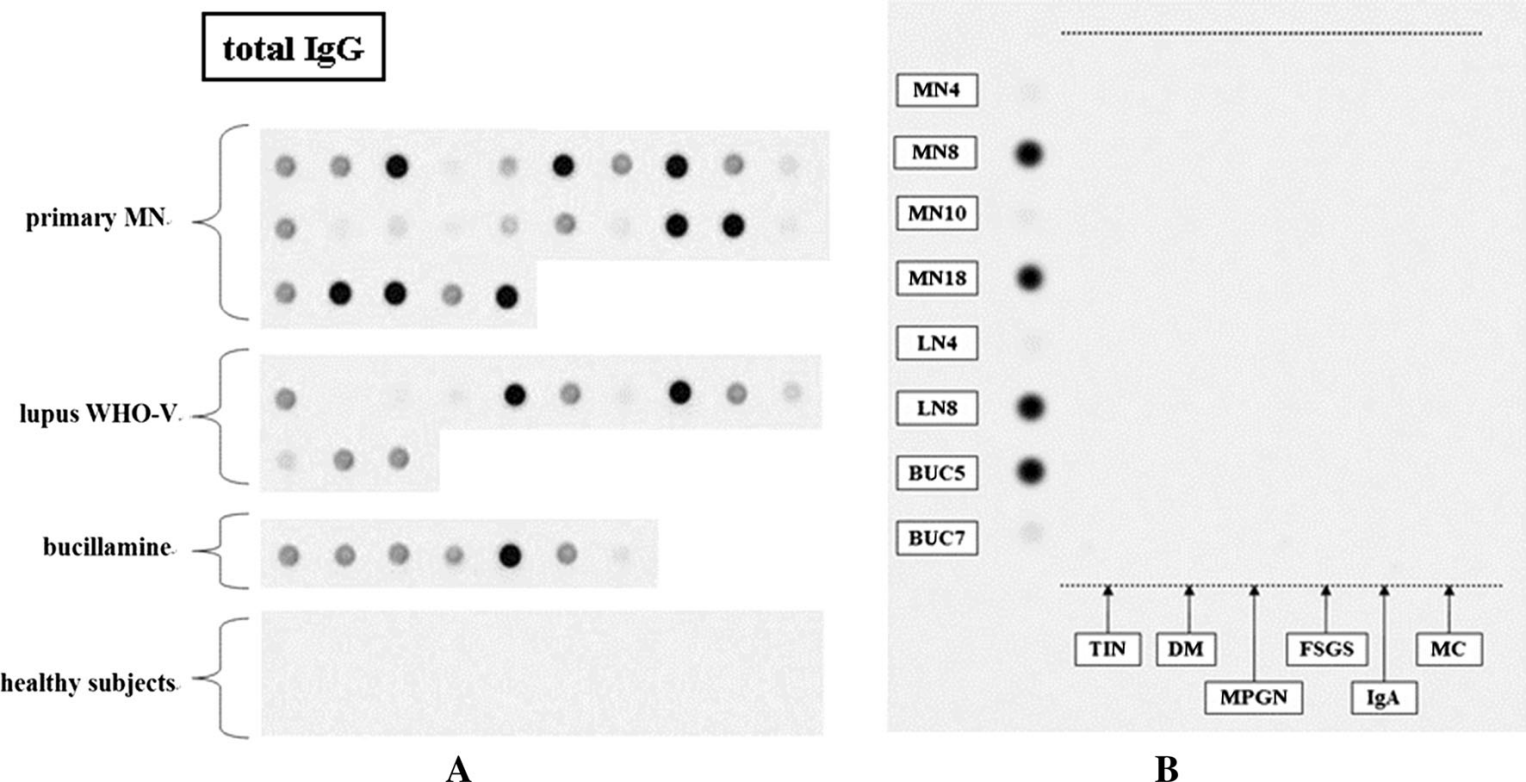
Immunoreactivity observed with full-length α-enolase in dot blot assays. **a** Sera from 18 of 25 patients (72 %) with primary MN immunoreacted with α-enolase. Nine of 13 patients with lupus nephritis (World Health Organization class V), (69 %) and 6 of seven patients with bucillamine-induced MN (86 %) were positive for anti-α-enolase antibodies. **b** *Left line* primary membranous nephropathy MN4 and MN10 were negative, and MN8 and MN 18 were positive. lupus nephritis LN4 and LN8 was negative and positive, respectively. bucillamine-induced MN BUC5 was positive and BUC7 was negative. *Right side lanes* included each ten patients with tubulointerstitial nephritis (TIN), diabetic glomerulosclerosis (DM), membranoproliferative glomerulonephritis (MPGN), focal segmental glomerulosclerosis (FSGS), IgA nephropathy (IgA), and minimal change nephrotic syndrome (MC), which were all negative

We first examined whether sera from patients with MN contained antibodies specific for recombinant α-enolase. Sera from 18 of 25 patients with primary MN (72 %) were immunoreactive with α-enolase. Nine of 13 patients with lupus nephritis (World Health Organization class V), (69 %) and six of seven patients with bucillamine-induced MN (86 %) were positive for anti-α-enolase antibodies (Fig. 1a). The inhibition tests showed highly specific antibody to α-enolase (*supplementary materials 1*). In contrast, sera from 44 collagen diseases or septic patients, 60 nephritis without MN as disease controls (Fig. 1b), and 20 healthy controls did not react with α-enolase.

### IgG subclasses of circulating anti-α-enolase antibodies

**Fig. 2:**
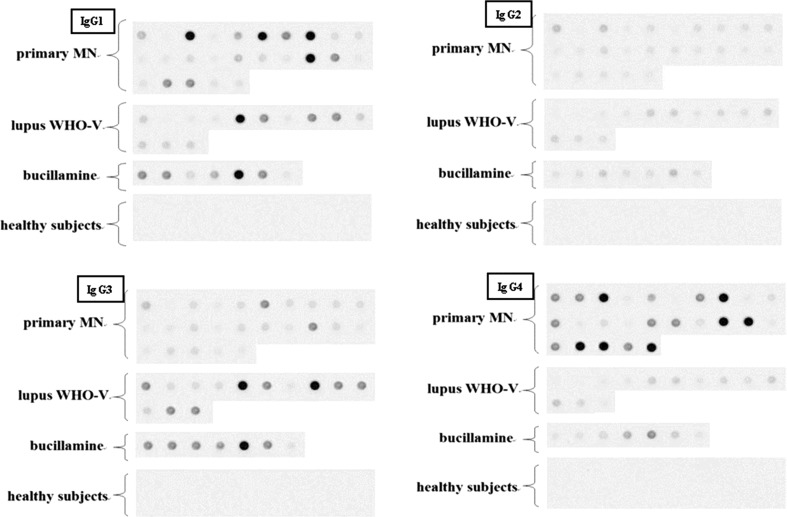
Circulating anti-α-enolase IgG antibody subclasses in primary and secondary MN. *Upper left* IgG1 subclass, *upper right* IgG2 subclass, *lower left* IgG3 subclass, *lower right* IgG4 subclass. For primary MN, most antibodies appeared to be IgG1 or IgG4; 72 % of patients were positive for at least one of these subclasses on dot blots. For secondary MN, however, IgG1 and IgG3 produced the strongest signals, with positive results observed in 75 % of patients

We then characterized IgG subclasses of anti-α-enolase antibodies in both primary and secondary MN (Fig. 2). In primary MN, most antibodies appeared to be IgG1 or IgG4; 72 % of patients being positive for at least one of these subclasses on dot blots. In secondary MN, however, IgG1 and IgG3 produced the strongest signals, with positive results observed in 75 % of patients, contrasting very little or absent IgG4 reactivity. Serum from each patient that immunoreacted with full-length α-enolase also was positive with the N- and C-terminal fragments (amino-acid stretch 1–66 and 349–433, respectively) (*supplementary materials 2*-*5*). Further to test whether the N- and C-termini have closely related epitopes, we blocked reactivity to the N-terminal fragment with the C-terminal fragment and conversely. The results suggest that the epitope/s at the both ends of protein are partially related. However, further studies are necessary to identify an exact epitope (or epitopes) recognized by circulating antibodies.

### Circulating anti-α-enolase antibodies before and after treatment

**Fig. 3:**
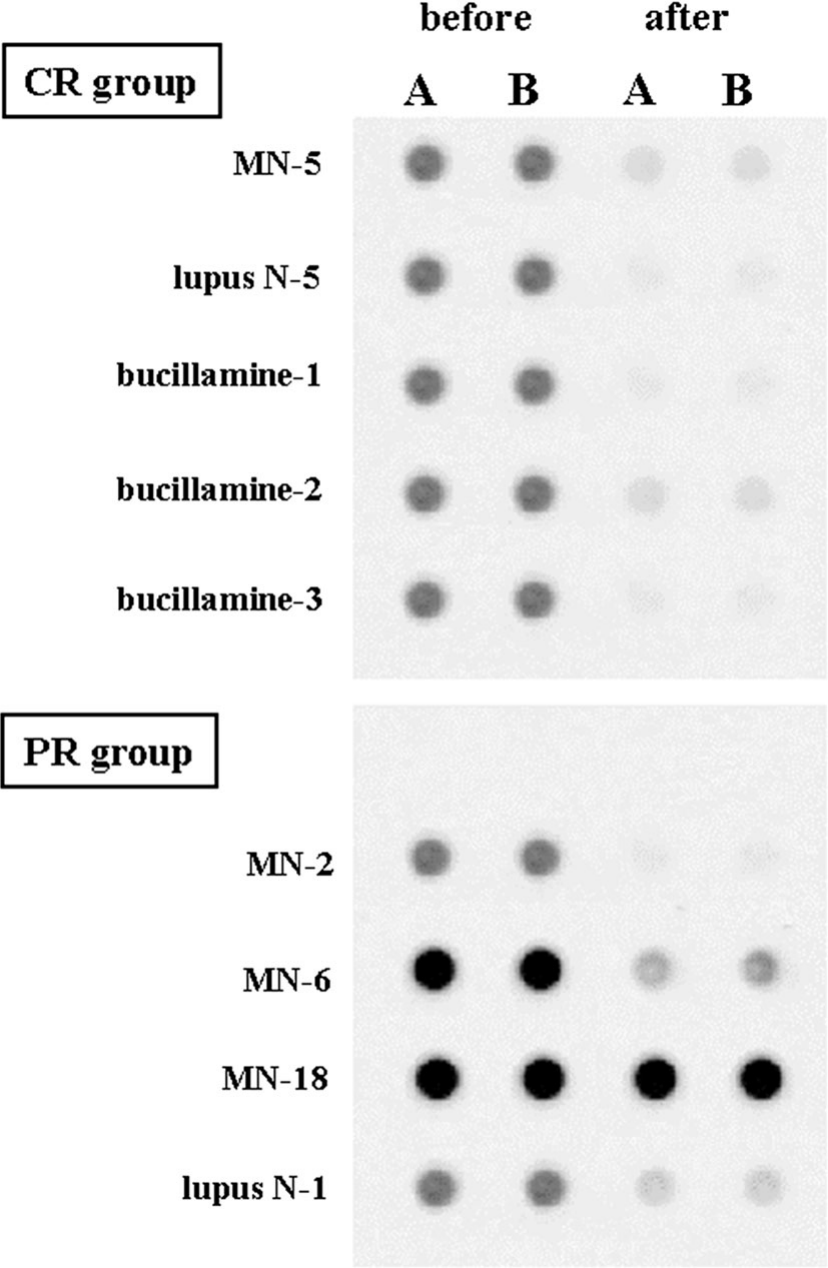
Circulating antibodies specific for α-enolase before and after treatment for MN. **a** The complete remission group contains sera from primary MN-5 and secondary MN (lupus nephritis-6, and bucillamine -1, -2, 3). In MN-5, signals for IgG1 and IgG4 became negative. Signals for IgG1 and IgG3 were no longer detected in all secondary MN patients after treatment. **b** The partial remission group contains sera from primary MN-2, -6, -18, and secondary MN (lupus nephritis-1). In MN-2, IgG4 antibody is still present, albeit with a reduced intensity. In MN-6, strong signals for IgG1 were shown before treatment, and remained unchanged after treatment. In MN-18, the signals for IgG1 and IgG4 were still positive, but with reduced intensity. In LN-1, antibodies of the IgG3 subclass were not observed after treatment. Lanes “f” and “g” are the 1142F and the 295R fragments of α-enolase, respectively

We examined the effects of treatment on circulating antibodies specific for α-enolase in a patient with primary MN, a patient with lupus MN, and three patients with bucillamine-induced MN, who all attained complete remission after treatment. We also assessed three patients with primary MN and one patient with lupus MN who failed to achieve complete remission despite therapy. Notably, circulating antibodies were no longer detected in each of the patients who reached complete remission (Fig. 3). Among the 4 patients who failed to attain complete remission, antibody titers were markedly decreased in the patient with lupus MN, and in two of three patients with primary MN, and unchanged in the remaining patient with prevailing IgG1 (Fig. 3).

### Anti-PLA_2_R antibody

Twelve of 25 (48 %) tested sera from patients with primary MN had anti-PLA_2_R antibodies (Table 1). Of the 12 PLA_2_R-positive patients, 8 were positive for anti-α-enolase, 4 were negative. Of the 13 PLA_2_R-negative patients, 10 were positive for anti-enolase. None of the 19 examined patients with secondary MN were positive for anti-PLA_2_R antibodies (Table 2).

### Glomerular deposition of PLA_2_R and α-enolase proteins

**Fig. 4:**
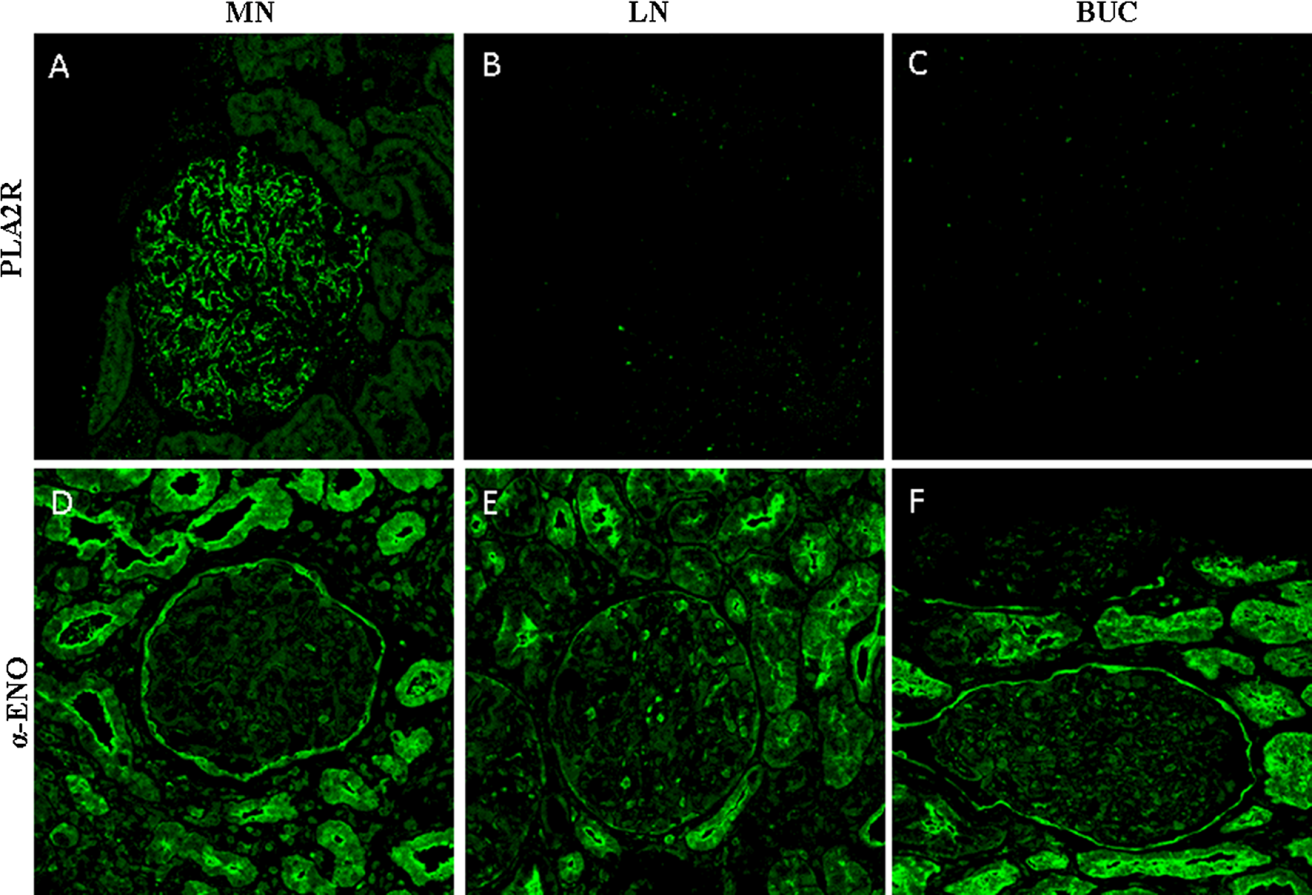
Glomerular deposition of PLA_2_R and α-enolase proteins in primary and secondary MN analyzed by confocal microscopy. PLA_2_R was present in primary MN (**a**), but not in secondary MN due to lupus nephritis (LN) and bucillamine (BUC) (**b**, **c**). α-enolase was strongly expressed in tubular epithelium and was weak positive in glomerular parietal cells (**d**, **e**, **f**), but was not detected in subepithelial deposits along glomerular capillary loops as was shown for PLA_2_R in primary MN

**Fig. 5:**
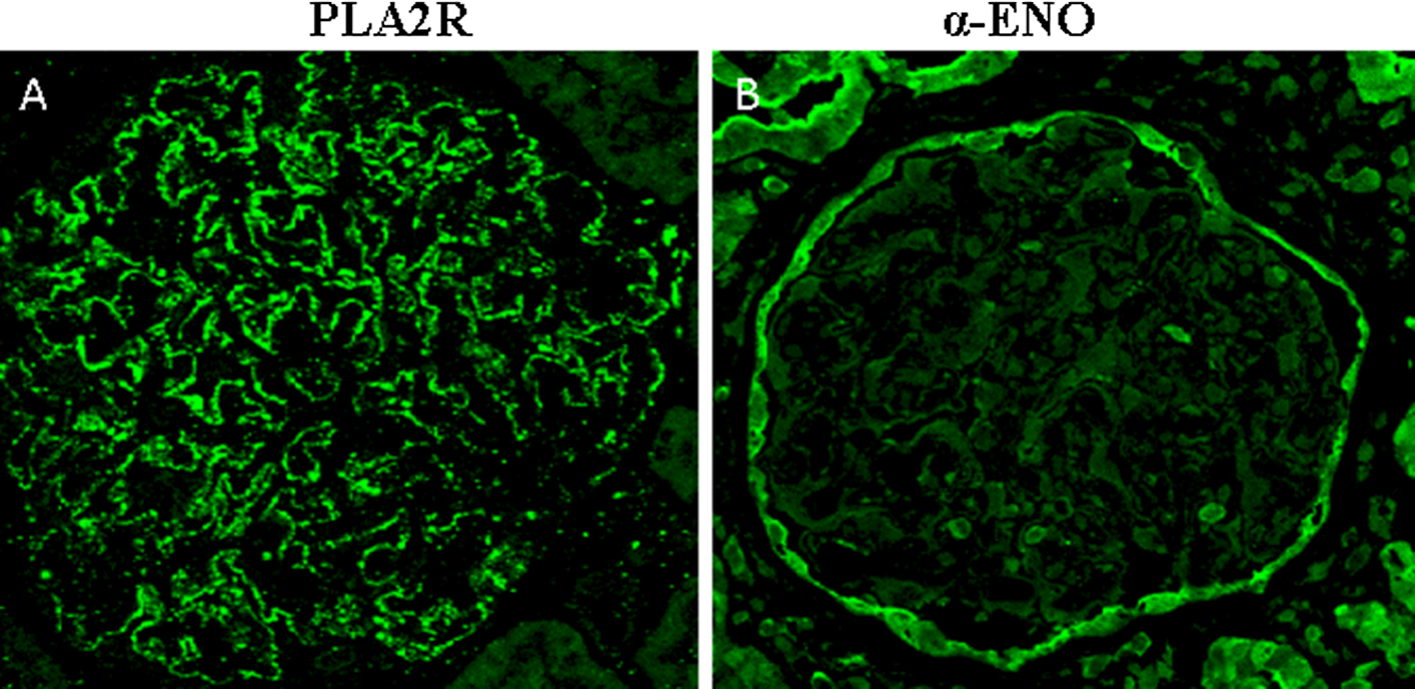
High magnification of PLA_2_R and α-enolase proteins in primary MN. PLA_2_R was detected in subepithelial deposits along glomerular capillary loops (**a**) whereas α-enolase was never detected in subepithelial deposits (**b**)

In paraffin-embedded kidney biopsy specimens, confocal microscopy showed the presence of PLA_2_R in subepithelial deposits along glomerular capillary loops (Figs. 4a, 5a) in 10 of 19 patients with primary MN. Nine of the ten patients also had circulating anti-PLA_2_R antibodies (Table 1). PLA_2_R was absent in the 10 examined biopsy specimens from patients with lupus- and bucillamine-related MN (Fig. 4b, c).

α-enolase was strongly expressed in tubular epithelium and was weak positive in glomerular parietal cells (Fig. 4d, e, f) and very weakly detected in the cytoplasm of glomerular cells on high magnification (Fig. 5b), but unlike PLA_2_R, α-enolase was never detected in subepithelial deposits in the 29 biopsy specimens where this was examined (Fig. 5b). The pattern of expression for α-enolase was the same in all groups, irrespective of the presence or absence of circulating anti α-enolase antibodies. Human IgG-adsorbed primary and secondary antibodies tested on frozen sections gave no signal in glomeruli but still well visible staining in proximal tubules (*supplementary materials 6*).

## Discussion

Our study provides important data on immunopathology of MN. It first shows that circulating anti-α-enolase IgG4 antibodies, with or without IgG1 antibodies, were present in 72 % of the patients with primary MN, while anti-α-enolase IgG3 antibodies, with or without IgG1 antibodies, were detected in 75 % of those with secondary MN. Circulating antibodies disappeared in the few tested patients undergoing complete remission. Second, we showed a low prevalence (48 %) of anti-PLA_2_R antibodies in this small cohort of Japanese patients with primary MN assessed before treatment, with eight of 25 patients producing both antibodies (32 %), while we could not detect anti-PLA_2_R antibodies in secondary MN such as bucillamine-induced MN where they had not been searched as yet. Third, we could not detect α-enolase in subepithelial immune deposits in any of the patient with circulating anti-α-enolase antibodies, which questions their role in the pathogenesis of MN, including PLA_2_R -unrelated MN, whereas PLA_2_R was detected in the deposits in all nine tested patients with anti-PLA_2_R antibodies and in one patient without circulating antibodies.

Anti-α-enolase antibodies have been reported in patients with a large variety of autoimmune and inflammatory diseases such as systemic lupus erythematosus (SLE), rheumatoid arthritis (RA), and autoimmune mediated retinopathy (AR) [22], with a prevalence of 21, 25 %, and about 30 %, respectively [23–25]. Based on our results and those from Wakui et al. approximately 70 % patients with primary or secondary MN carry anti-α-enolase antibodies [13]. We detected mostly IgG4 and IgG1 anti-α-enolase antibodies in primary MN, and IgG3 and IgG1 anti-α-enolase antibodies in secondary MN, while Wakui et al. predominantly identified IgG1 and IgG3 anti-α-enolase antibodies. These discrepancies might be due to different affinities of the anti-subclass antibodies used for detection. More recently, Bruschi et al. found that anti-α-enolase IgG4 antibody levels were high in only 25 % of patients with primary MN [14]. The different prevalence of anti-α-enolase antibodies between Japanese and Italian populations may reflect underlying genetic factors, including human leukocyte antigen polymorphisms. Although anti α-enolase antibodies were reported to be detected in sera of healthy subjects [26, 27], we could not detect anti-α-enolase antibodies in disease controls including those with connective disease like Bruschi et al. [14]. One possible explanation of this discrepancy might be the technical approaches and their different sensitivity and specificity.

Because of the apparently wide spectrum of diseases associated with anti-α-enolase antibodies, these antibodies by themselves cannot help for the diagnosis of specific autoimmune diseases. Therefore determination of immunoreactive sequences of α-enolase associated with disease-specific pathogenic autoantibodies is of great importance. Epitope mapping performed in cancer-associated retinopathy (CAR) showed the epitope located between amino-acids 56 and 63 (56–63aa) being specifically associated with pathogenic sera [28]. In endometriosis, anti- α-enolase antibodies bound to two epitopes at 53–87aa and 207–238aa and shared reactivity against the epitope 56–63aa with CAR patients [29]. The epitope for pathogenic antibodies in Hashimoto’s encephalopathy is located in the N-terminal part of a-enolase protein [30]. In our patients, antibodies bound to epitopes located in the first 66aa at the N-terminal end and in the last 84aa at the C-terminal end of the protein. The next step will be to perform fine epitope mapping of these regions, and to investigate the pathogenic relevance of these epitopes.

α-Enolase is an ubiquitous cytoplasmic glycolytic enzyme involved in the synthesis of pyruvate [31]. In addition to its glycolytic function, this 47-kDa glycoprotein exerts many other functions related to its subcellular location [31]. Different cellular localizations of potential MN auto-antigens are seen under normal physiologic conditions. While neutral endopeptidase and PLA_2_R are expressed on cell surface of podocytes [9, 11, 32], aldose reductase and SOD2 are cytoplasmic/mitochondrial enzymes which can be enhanced at the cell surface after oxidative stress [12]. Similarly, α-enolase is usually cytoplasmic but its expression varies according to the cell conditions and can be expressed at the surface of many eukaryotic cells [31].

Although circulating anti-α-enolase antibodies were commonly found, unlike PLA_2_R, α-enolase was never detected in subepithelial immune deposits, in contrast with the observations made by Bruschi et al. [14]. We used human IgG-adsorbed primary and secondary antibodies tested on frozen sections gave no signal of α-enolase in glomeruli but still well visible staining in proximal tubules. This finding suggests that anti-α-enolase antibody does not directly contribute to the formation of subepithelial immune deposits and resulting complement activation. We cannot, however, exclude a pathogenic role for anti-α-enolase antibodies through binding to endothelial or podocyte cell surface, and subsequent alteration of cell functions. Alterations induced by binding of anti-α-enolase antibodies to the surface of endothelial cells or podocytes may increase permeability of the capillary wall, leading to enhanced access and accumulation of pathogenic antibodies such as anti- PLA_2_R. Furthermore, because α-enolase has pleiotropic effects, antibodies may perturb other important cellular functions. For instance, anti-α-enolase antibodies were shown to inhibit the binding of plasminogen to α-enolase on cell surface, which means protective effects from proteolytic activities of plasmin [33, 34]. On the contrary, intracellular anti-α-enolase antibodies taken-up by endocytosis may interfere with glycolysis, decrease cellular ATP levels, and increase intracellular Ca^2+^, which ultimately may induce apoptosis [35].

We found that the prevalence of anti-PLA_2_R antibodies in previously untreated patients (48 %) was lower than that previously reported in Caucasian, African, and Chinese cohorts [11, 36–38], suggesting an effect of the genetic background. Akiyama et al. reported similar data that 53 % of patients with idiopathic MN in Japan [39].

In conclusion, circulating anti-α-enolase antibody was detected in about 70 % of patients with both primary and secondary MN, while anti-PLA_2_R antibody was restricted to primary MN. The absence of glomerular deposition of α-enolase in subepithelial area contrasting with the presence of PLA_2_R in immune deposits, suggests that instead of being implicated in the formation of immune deposits, anti-α-enolase antibody binding to glomerular cell surface might increase access to the podocyte of other antibodies such as anti-PLA2R, and thus be an enhancing event in primary and secondary MN. Further studies on larger cohorts are needed to confirm this hypothesis and to delineate the value of anti-α-enolase antibody as biomarker for diagnosis and monitoring of MN patients and of α-enolase as potential therapeutic target.

## Electronic supplementary material

Below is the link to the electronic supplementary material.

Supplementary material 1 (GIF 25 kb)

Supplementary material 2 (GIF 75 kb)

Supplementary material 3 (GIF 41 kb)

Supplementary material 4 (GIF 99 kb)

Supplementary material 5 (GIF 94 kb)

Supplementary material 6 (GIF 69 kb)
