# Supplementary figures and images for: Circulating antibodies to α-enolase and phospholipase A2 receptor and composition of glomerular deposits in Japanese patients with primary or secondary membranous nephropathy
Source: Clin Exp Nephrol. 2016 Feb 1;21(1):117–26. doi: 10.1007/s10157-016-1235-2 (PMC5283514; doi:10.1007/s10157-016-1235-2)

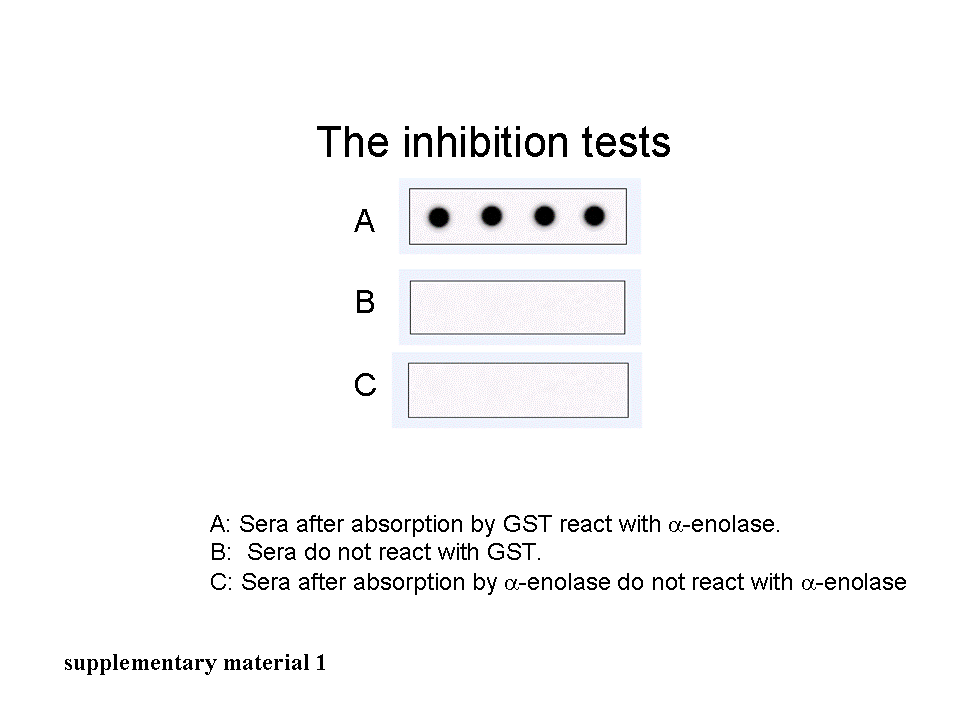

Supplement: Supplementary file 1 — Supplementary material 1 (GIF 25 kb) [file 10157_2016_1235_MOESM1_ESM.gif]

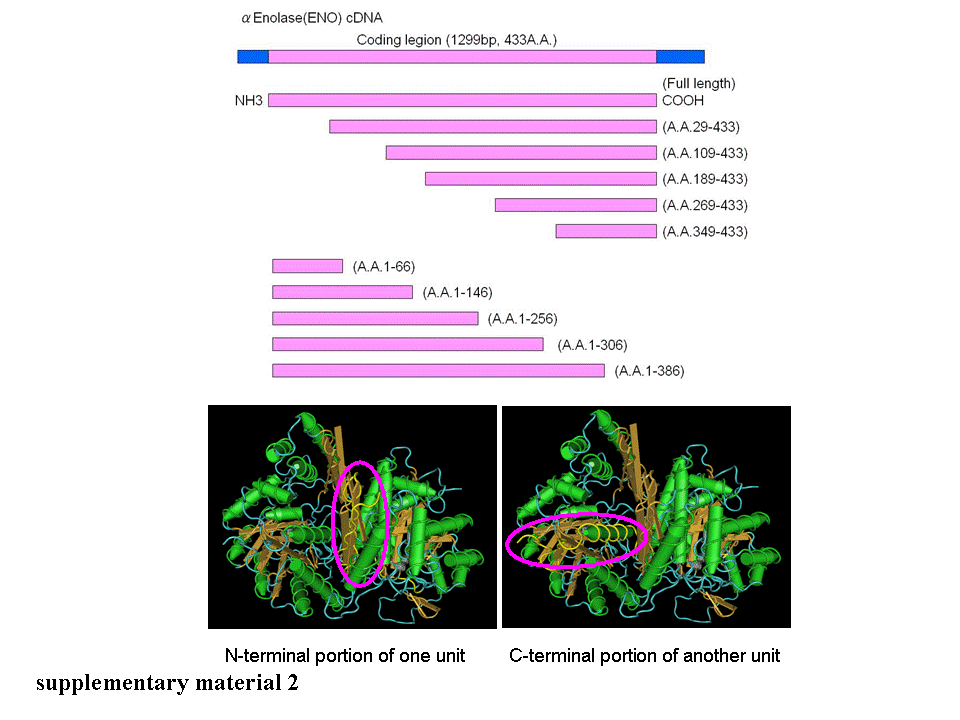

Supplement: Supplementary file 2 — Supplementary material 2 (GIF 75 kb) [file 10157_2016_1235_MOESM2_ESM.gif]

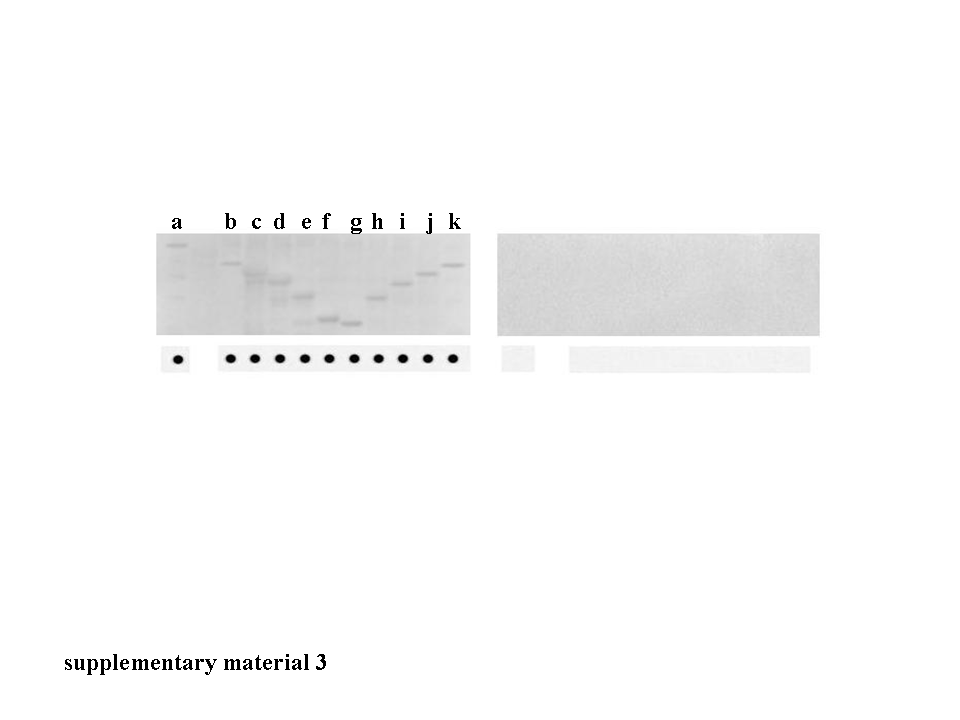

Supplement: Supplementary file 3 — Supplementary material 3 (GIF 41 kb) [file 10157_2016_1235_MOESM3_ESM.gif]

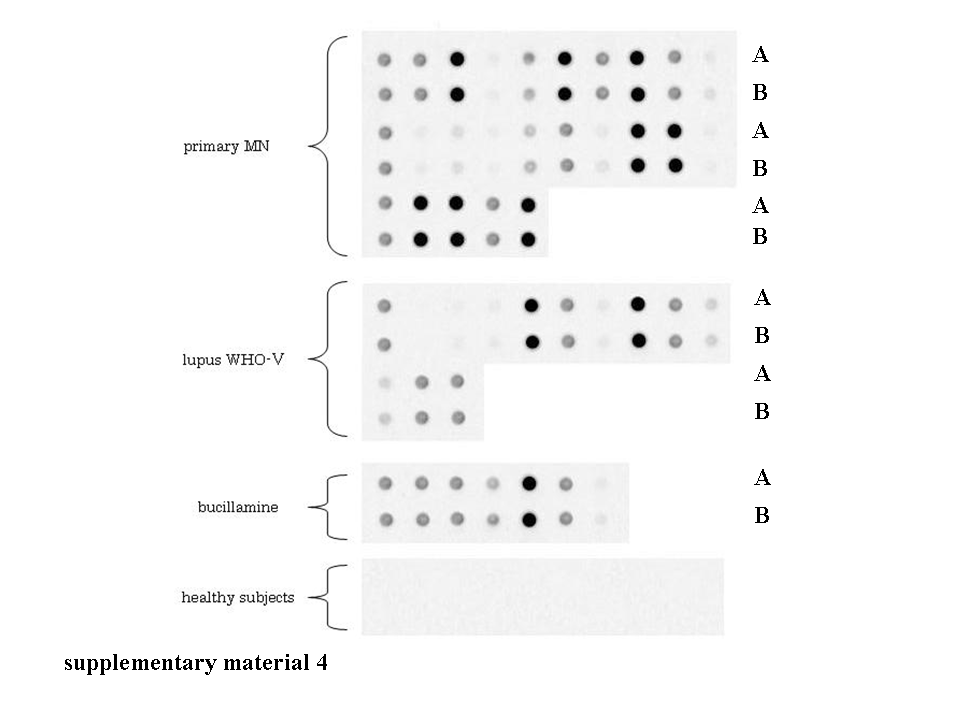

Supplement: Supplementary file 4 — Supplementary material 4 (GIF 99 kb) [file 10157_2016_1235_MOESM4_ESM.gif]

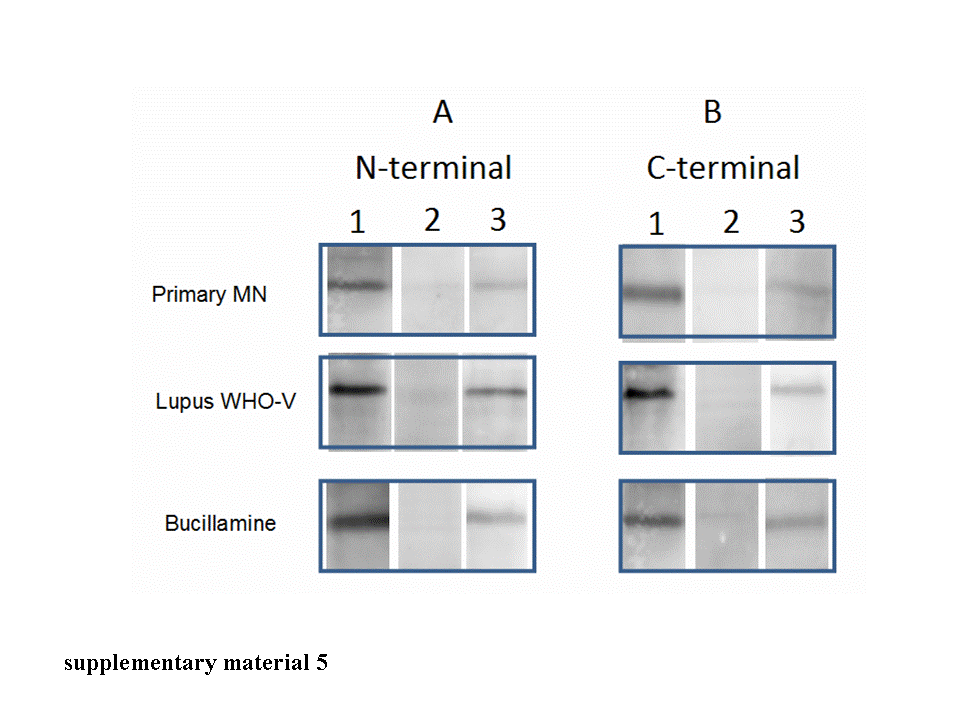

Supplement: Supplementary file 5 — Supplementary material 5 (GIF 94 kb) [file 10157_2016_1235_MOESM5_ESM.gif]

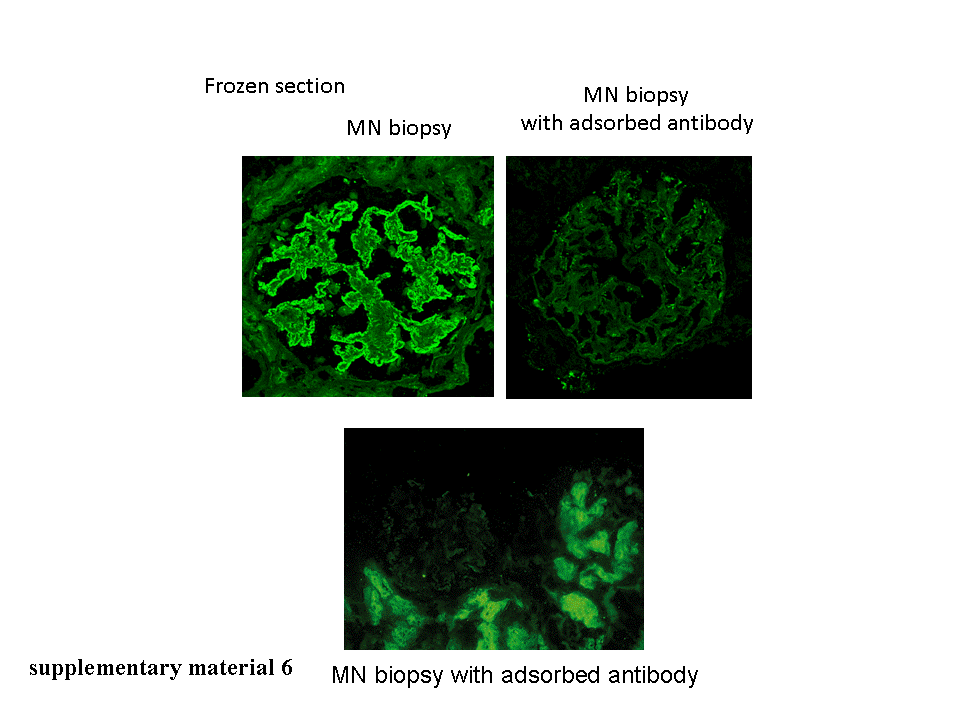

Supplement: Supplementary file 6 — Supplementary material 6 (GIF 69 kb) [file 10157_2016_1235_MOESM6_ESM.gif]
